# Effect of Ambient Pressure and Pulse Shaping on Femtosecond Laser Drilling of GDP Films

**DOI:** 10.3390/ma19173665

**Published:** 2026-08-28

**Authors:** Qinxin Wang, Xinyue Zhang, Zhan Hu

**Affiliations:** 1 School of Data Science and Artificial Intelligence, Jilin Engineering Normal University, Changchun 130052, China; zhangxyy0304@163.com; 2 Institute of Atomic and Molecular Physics, Jilin University, Changchun 130012, China

**Keywords:** femtosecond laser, ambient pressure, GDP film, microhole drilling, pulse shaping, plasma shielding, processing efficiency

## Abstract

The quality and precision of polymer film processing via femtosecond laser are frequently degraded by the redeposition of ablated material. Here, we systematically investigate the effects of backing pressure and temporal pulse shaping on the drilling of glow discharge polymer (GDP) films. Reducing the pressure effectively suppresses debris redeposition, improves surface cleanliness, and enhances drilling efficiency. The entrance diameter exhibits a non-monotonic pressure dependence, with a minimum near 10^3^ Pa, whereas the exit diameter decreases monotonically as pressure increases. At high pressures, through-hole formation becomes increasingly difficult, particularly for pulse sequences with a larger number of subpulses. Low pressure substantially reduces the pulse count required for breakthrough. The observed behavior is interpreted in terms of pressure-dependent nonlinear propagation, focal-position shifts, and plasma shielding. By combining low-pressure operation with an optimized pulse sequence, we obtain high-quality microholes with reduced taper and improved drilling efficiency. These results provide practical guidance for precision femtosecond laser micromachining of polymer films.

## 1. Introduction

Femtosecond laser micromachining has become a widely adopted technique for high-precision material processing, as ultrashort pulses confine energy deposition both temporally and spatially, thereby limiting thermal diffusion and collateral damage [1,2,3,4,5,6,7,8]. This capability makes femtosecond irradiation particularly suitable for fabricating micro- and nanoscale structures, especially in transparent materials [5,6,7,8]. Glow discharge polymer (GDP) is a transparent hydrocarbon polymer with favorable optical uniformity, mechanical stability, and chemical resistance [9,10,11]. It is employed in applications such as inertial confinement fusion targets, microfluidic devices, and microelectromechanical systems, where microholes with well-controlled dimensions, clean surfaces, and low taper are often required [9,12,13,14]. However, producing such structures in GDP films remains challenging, as the final hole geometry is governed by coupled laser–material interaction, plasma dynamics, and subsequent material transport processes [15,16,17,18,19,20].

During femtosecond laser drilling, a broad distribution of atoms, ions, clusters, molten droplets, and large chunks of debris are formed. The transport and evolution of these ablation products are strongly influenced by the surrounding gas environment [20,21,22,23]. At elevated pressures, frequent collisions between the ablation products and ambient gas molecules shorten the mean free path of the ejected species and confine the expanding plume. This confinement decelerates and cools the ejected material, promoting its return toward the interaction region, which in turn increases redeposition, contaminates the surface, and degrades the edge quality of the hole [20,21,22,23,24,25,26]. Pressure-dependent modifications to hole morphology and laser focusing have been reported in femtosecond drilling of metals, highlighting the role of the gas environment in determining the final microstructure [21,27]. Ambient pressure thus serves as a critical control parameter for plume expansion, debris redeposition, and beam propagation [20,21,22,23,24,25,26,27]. However, the influence of the gas environment on laser ablation of GDP materials still requires further investigation. In particular, identifying the optimal pressure that enhances the quality and precision of polymer film processing would be of great advantage for practical applications. In addition, laser pulse shaping offers a complementary degree of freedom: by redistributing pulse energy temporally, it can modify excitation dynamics, plasma evolution, and the interaction of subsequent subpulses with the evolving ablation region [28,29,30,31,32,33,34]. Despite these advances, the combined influence of ambient pressure and pulse shaping on femtosecond laser drilling of GDP films has not been systematically established. In particular, the coupled pressure dependences of entrance and exit diameters, hole taper, and drilling efficiency—along with the roles of self-focusing, focal-position shifts, and plasma shielding—require further clarification [9,35,36,37].

In this work, we investigate femtosecond laser drilling of GDP films over a broad range of ambient pressures using both transform-limited pulses and shaped pulse sequences. We analyze the pressure dependences of hole morphology, entrance and exit diameters, taper, and breakthrough pulse count, and we develop a consistent physical interpretation based on pressure-dependent nonlinear propagation, plume confinement, and plasma shielding. The results provide both fundamental insight and practical guidance for high-quality, high-efficiency micromachining of polymer films.

## 2. Materials and Methods

**Figure 1:**
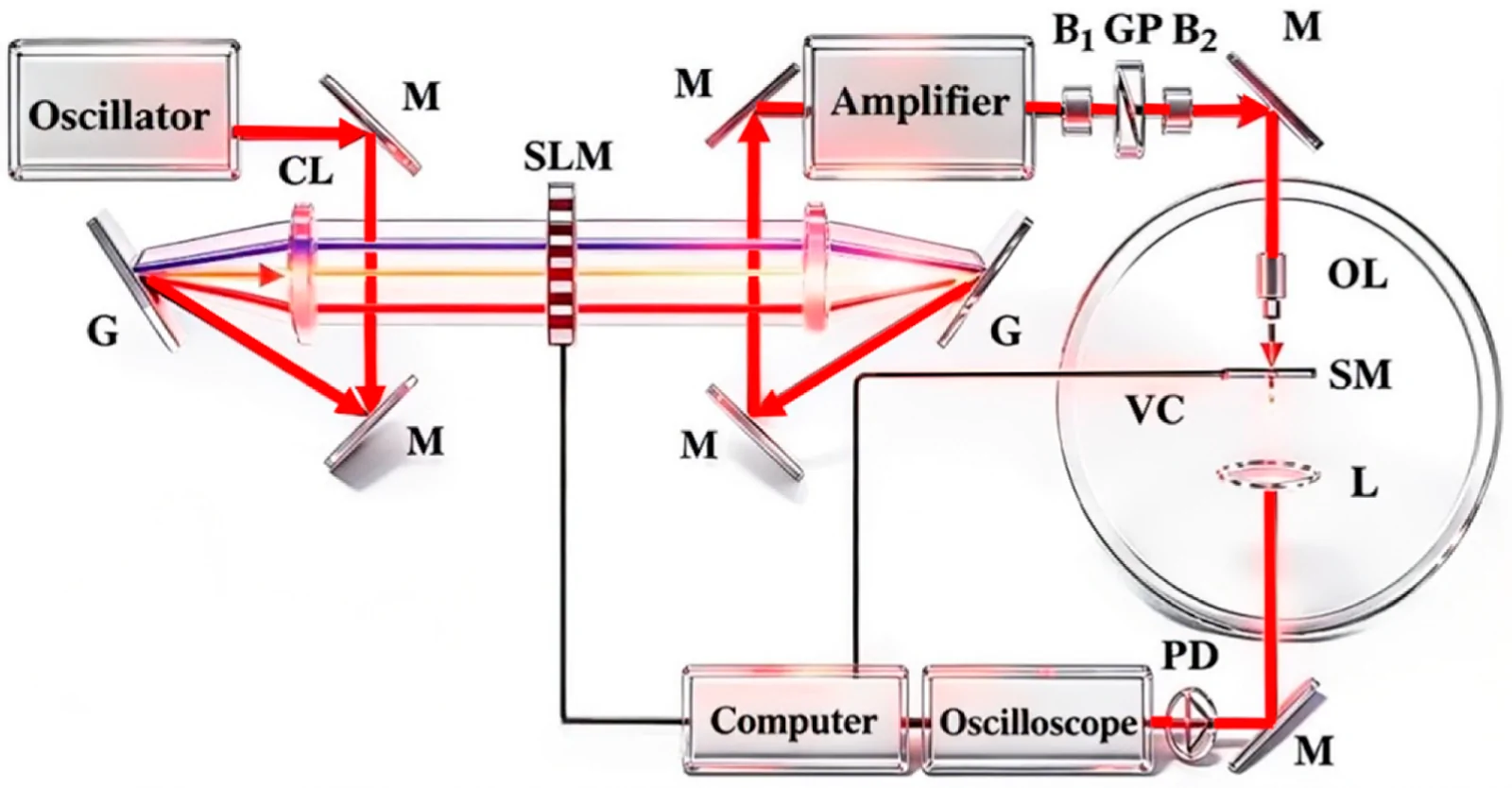
Shaped-femtosecond laser vacuum ablation system [9]. G, grating; CL, cylindrical lens; SLM, spatial light modulator; M, mirror; GP, Glan prism; B_1_/B_2_, wave plates; VC, vacuum chamber; OL, objective; SM, sample; L, lens; PD, photodiode.

The experiments were conducted using a femtosecond laser micromachining system integrated with a vacuum chamber. The system consisted of a Ti:sapphire femtosecond laser source (Spectra-Physics Spitfire, 1 kHz) with a central wavelength of 800 nm, a chirped-pulse amplification module, a 640-pixel dual-mask spatial light modulator (SLM, CRI SLM-640-D-VN) for temporal pulse shaping in a standard 4-f configuration, a beam-delivery and focusing system, and a three-axis motorized translation stage (Newport 426) for sample positioning as shown in Figure 1 (Doubao was employed to refine the setup figure with our input layout) [9,33]. The temporal profiles of the laser pulses were controlled via inputting the designed phase patterns applied to the SLM through rotating the liquid crystal with the electric field, coded using a computer [38,39]. The laser delivered 90 fs pulses with a maximum pulse energy of 6.0 μJ at the sample. The temporal profiles of the laser pulses, controlled via phase functions applied to an SLM, were adjusted using a computer. Multipulse shaping was achieved by imposing a sinusoidal phase function on the laser spectrum, described by [9]

(1) $$\Phi =A\sin\left[\frac{2\pi }{T}\left(n-n_{0}\right)+\phi \right]$$

where A is the amplitude of the phase modulation, T is the period, n and n_0_ are the pixel positions on SLM, and ϕ determines the phase position on SLM. Three pulse configurations were investigated: (i) transform-limited single pulses where no phase modulation was applied to the spectrum, (ii) three-pulse sequences by setting A = 1.2566, and (iii) five-pulse sequences where A = 2.8276. For the shaped pulse sequences, the subpulse interval was fixed at 0.3 ps. Successive three or five subpulses will interact with the material during the early evolution of the laser-interaction region [9]. The laser beam was focused perpendicularly onto the sample through a microscope objective (Edmund NT59-877, 10×). The focused beam characteristics, including the beam diameter (1.96 µm) and Rayleigh length (24.2 µm), were quantified using the knife-edge method. The lens-to-sample geometry—and hence the nominal focal-plane position relative to the sample surface—was kept fixed while the ambient pressure was varied. This approach enables one to shift the focal position relative to the sample surface, by the pressure-dependent nonlinear propagation effect [40,41].

The samples were transparent GDP films with a thickness of 120 μm, mounted on a translation stage in a vacuum chamber where the ambient pressure could be adjusted from approximately 1 Pa to 1 atm. The GDP films are fabricated using plasma-enhanced chemical vapor deposition under varying tetramethylsilane (TMS) flow pressures. In this process, a gas mixture of TMS, trans-butene, and hydrogen is ionized within a quartz tube by a plasma generated from a radio-frequency power source, resulting in the deposition of GDP films. Following deposition, all films undergo in situ treatment with hydrogen plasma to further modify their properties. The produced GDP film is free-standing, mounted on the translation stage for further laser processing. During drilling, the laser beam remained stationary at each processing site, and the number of incident pulses was increased until breakthrough occurred. Drilling efficiency was evaluated from the number of pulses required to penetrate the film through the signal of photon intensity collected by a photodiode and oscilloscope, with a lower pulse count corresponding to higher efficiency. The fabricated microholes were characterized by optical microscopy (Olympus BX51) after interaction. The entrance and exit diameters were measured from the recorded images, and the entrance–exit diameter difference was used as a practical measure of hole taper.

**Figure 2:**
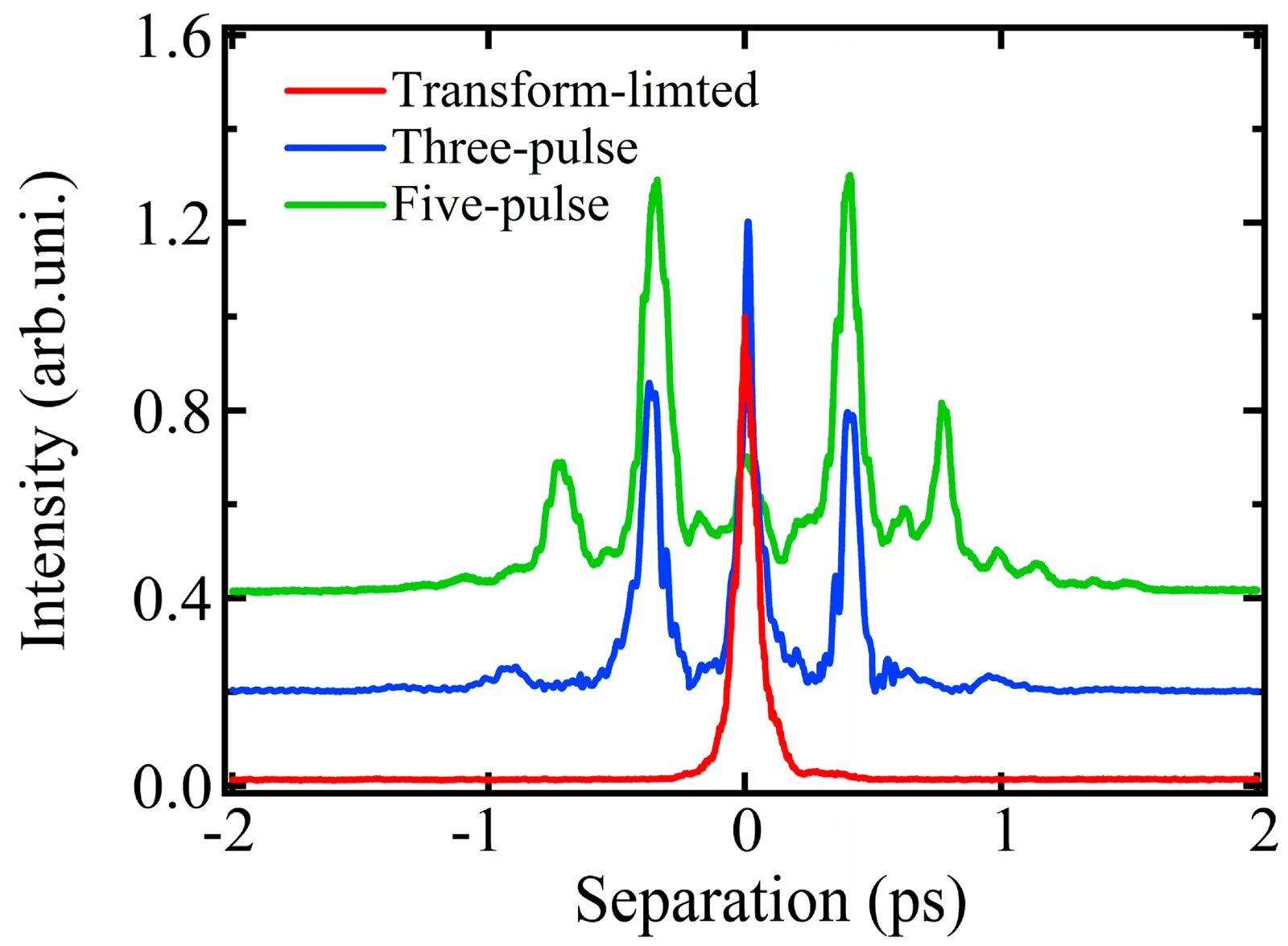
Cross-correlation measurement results of the femtosecond pulse, the transform-limited pulse (red), the shaped three-pulse profile (blue) and the corresponding five-pulse profile (green).

To characterize the temporal profiles of the transform-limited pulse and the shaped pulse trains, we performed cross-correlation measurements, with the results presented in Figure 2. The pulse shaping was achieved by applying a sinusoidal phase modulation to the laser spectrum via an SLM; we generated two distinct pulse sequences by selecting A = 1.2566 and 2.8274. Figure 2 shows the reference TL pulse (red curve). For A = 1.2566, the train consists primarily of three subpulses (blue curve), while for A = 2.8274, it contains five subpulses (green curve). During the experiment, the delay Δt was scanned from −2 ps to 2 ps in steps of 0.02 ps by varying separation between measured pulses and the references pulse (transform-limited pulse). The subpulse intervals for both shaped sequences were fixed at 0.3 ps, ensuring that successive subpulses interacted with the material during the early evolution of the laser–matter interaction region. All the sub-pulses are resolved in this cross-correlation measurement, and can be used to understand the time–energy distributions of shaped laser pulses.

## 3. Results and Discussion

### 3.1. Effect of Ambient Pressure on Hole Morphology

**Figure 3:**
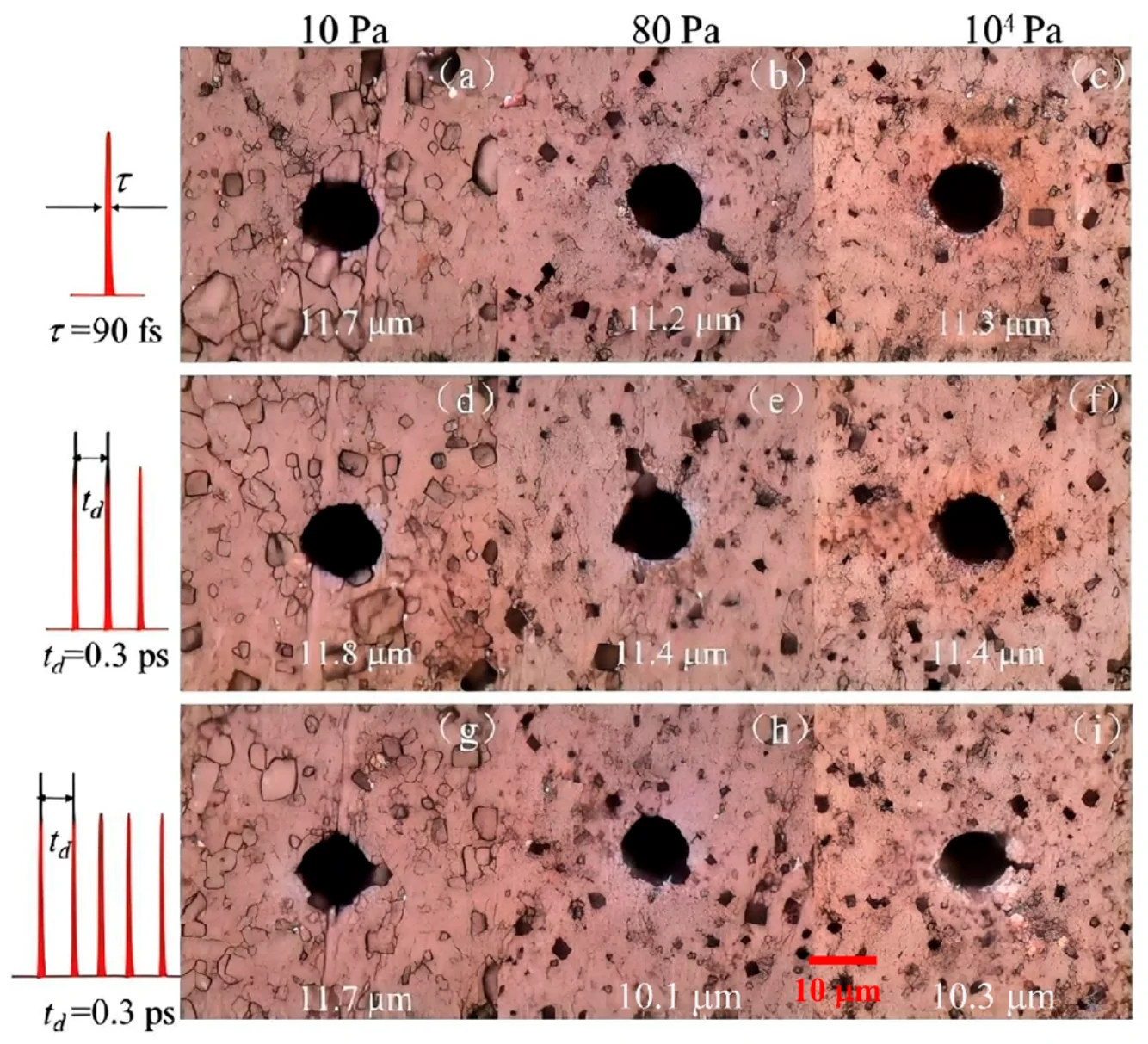
Optical micrographs of the entrance surfaces of microholes drilled at three selected ambient pressures (10 Pa, 80 Pa, and 10^4^ Pa) using transform-limited pulses (**a**–**c**), three-pulse sequences (**d**–**f**), and five-pulse sequences (**g**–**i**).

We varied the ambient pressure and compared the morphologies of the fabricated microholes. Selected optical micrographs of microholes drilled at different ambient pressures using transform-limited pulses and shaped pulse sequences are shown in Figure 3. The surface morphology exhibits a strong pressure dependence. At high pressure (~10^4^ Pa), the surrounding surface near the hole entrance shows pronounced discoloration, indicating extensive redeposition. This behavior is consistent with the higher gas density, which increases the collision frequency between ablation products and ambient molecules, slows plume expansion, and promotes the return and redeposition of ejected material [30,31,32,33]. When the pressure is reduced to an intermediate level (~80 Pa), the amount of redeposited debris decreases markedly, the surrounding surface becomes cleaner, and the hole edge is more clearly defined. At low pressure (~10 Pa), the entrance exhibits a sharp, well-defined edge with minimal visible debris. The longer mean free path under these conditions allows ablation products to expand away from the surface with weaker collisional confinement and a lower probability of returning to the processing region. Thus, over the investigated pressure range, the observations demonstrate a clear reduction in redeposition as the ambient pressure is lowered. This result confirms that low-pressure operation is an effective strategy for improving surface cleanliness during femtosecond laser drilling of GDP films.

**Figure 4:**
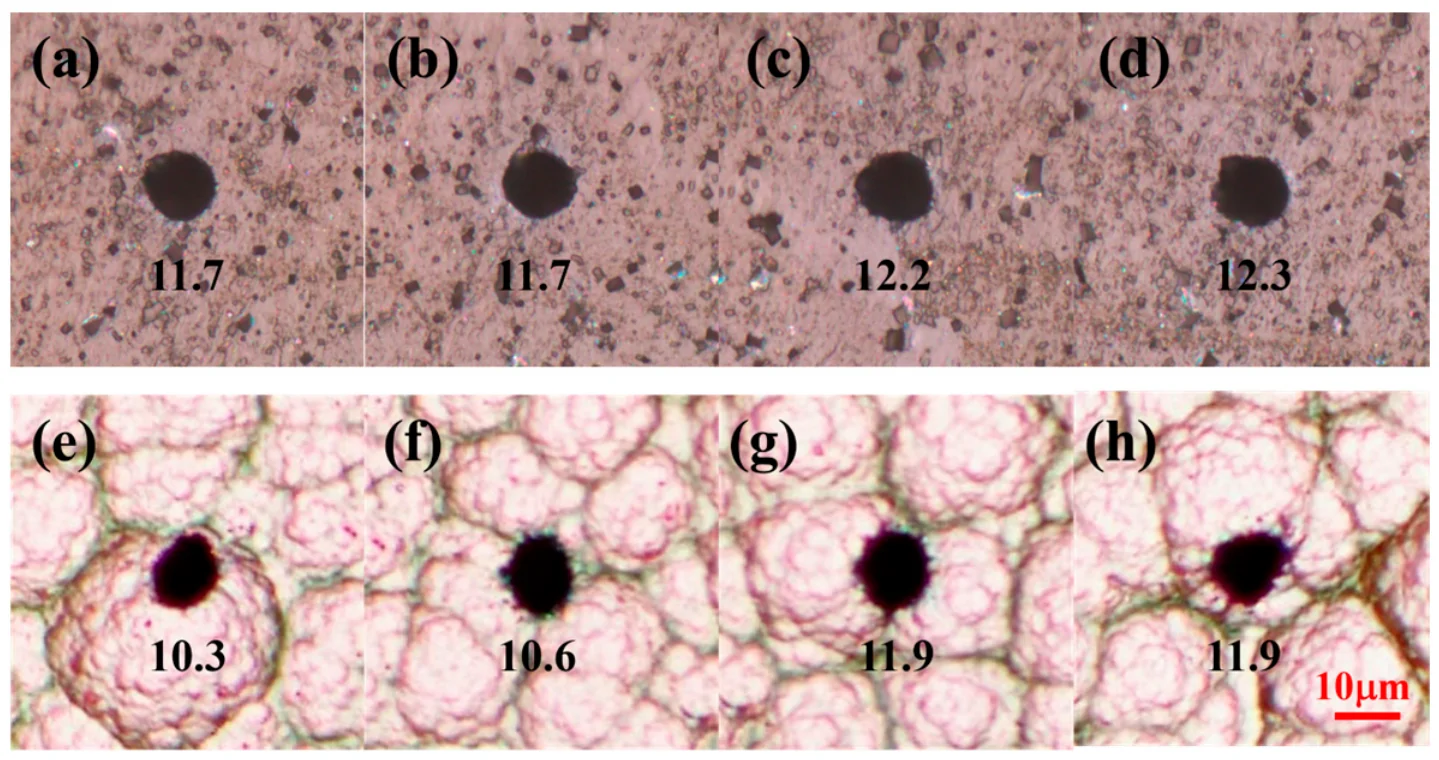
Optical micrographs of the entrance (**a**–**d**) and exit (**e**–**h**) of microholes drilled at ambient pressure of 10 Pa, changing the number of laser shots during drilling (3 × 10^4^, 4 × 10^4^, 5 × 10^4^, 6 × 10^4^ shots).

To further investigate the role of laser–material interaction on hole formation, we fixed the ambient pressure at 10 Pa, and systematically varied the number of laser shots applied during drilling. Figure 4a–d present optical micrographs of the hole entrances for the laser shots of 3 × 10^4^, 4 × 10^4^, 5 × 10^4^, and 6 × 10^4^, respectively, while Figure 4e–h show the corresponding exit morphologies. At the lowest shot number (3 × 10^4^), the entrance appears relatively small and irregular, and the exit is partially open, indicating incomplete penetration through the GDP film. As the shot number increases to 5 × 10^4^ shots, both the entrance and exit exhibit well-defined, clean contours with minimal taper, implying that steady-state ablation has been achieved and that the through-hole is fully developed with little collateral damage. Beyond this point, at 6 × 10^4^ shots, the entrance shows slight enlargement and the exit aperture broadens, which may be attributed to cumulative heat accumulation effects despite the low ambient pressure. These results indicate that while low pressure effectively mitigates redeposition, the shot number must be carefully optimized—at approximately 5 × 10^4^ shots for the present conditions—to balance complete penetration against excessive material removal or thermal side effects.

### 3.2. Hole Diameter Evolution Under Different Pressures

**Figure 5:**
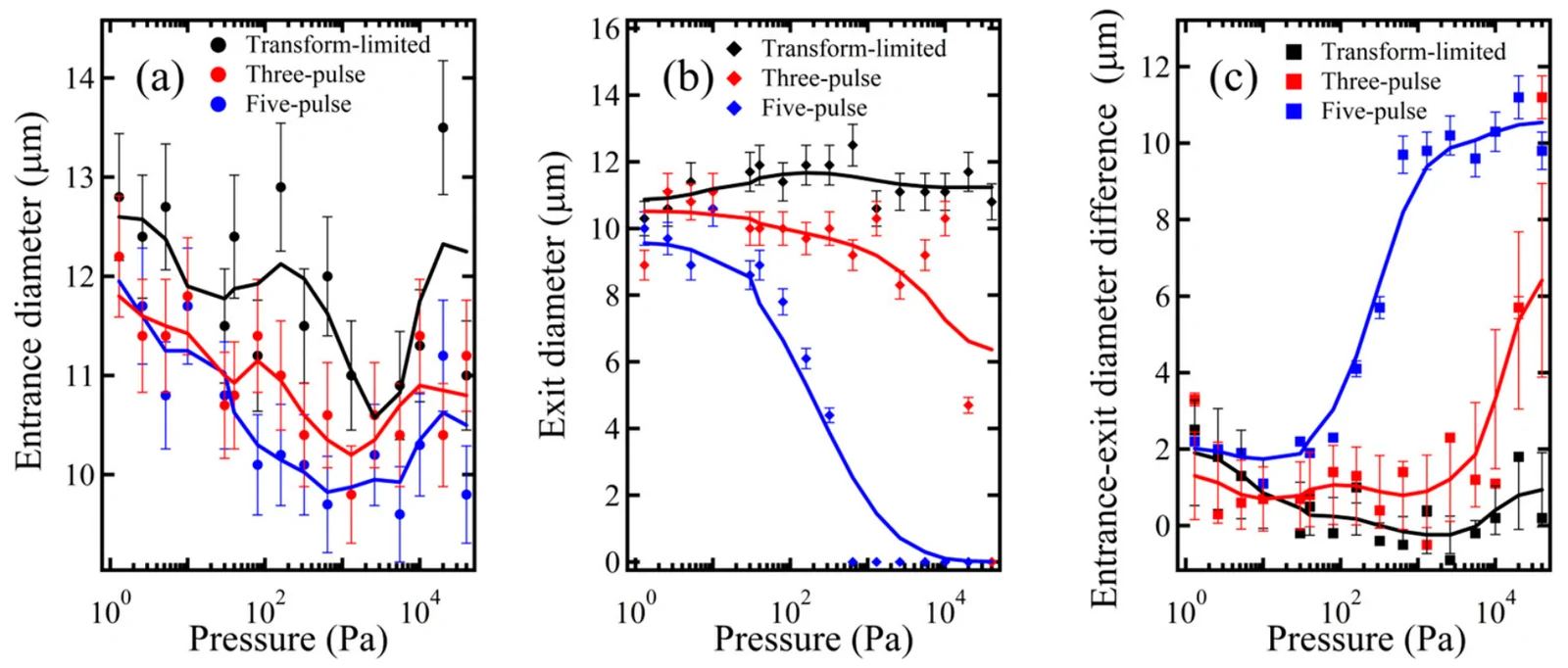
Pressure dependence of (**a**) entrance diameter, (**b**) exit diameter, and (**c**) entrance–exit diameter difference acquired under three different pulse shapes. All measurements were performed six times under each condition, and the trend lines serve as guides for the eye and were generated using Igor Pro 6.04 software.

Figure 5 presents the entrance diameter, exit diameter, and their difference as functions of backing pressure in the vacuum chamber for the three different pulse shapes. The entrance diameter shows a non-monotonic pressure dependence: it initially decreases with increasing pressure, reaches a minimum near 10^3^ Pa, and then increases at higher pressures. The exit diameter, in contrast, decreases monotonically with increasing pressure. Similar pressure-dependent changes in hole diameter and drilling behavior have been observed in femtosecond processing under controlled gas environments [21,22,23,24]. For the five-pulse sequence, the exit diameter approaches zero above ~4 × 10^2^ Pa, where breakthrough is not achieved under these conditions. The entrance–exit diameter difference also depends strongly on both pressure and pulse configuration. The transform-limited pulse and the three-pulse sequence yield comparatively small diameter differences over a broad pressure range, in which holes made by the three-pulse sequence have a relatively small diameter. For example, at low pressure (2.8 Pa), under the irradiation of the three-pulse sequence, the entrance–exit hole diameter difference is approximately 0.3 μm, with an entrance diameter of 11.3 mm and an exit diameter of 11.0 mm, corresponding to a highly uniform through-hole geometry with very low taper.

**Figure 6:**
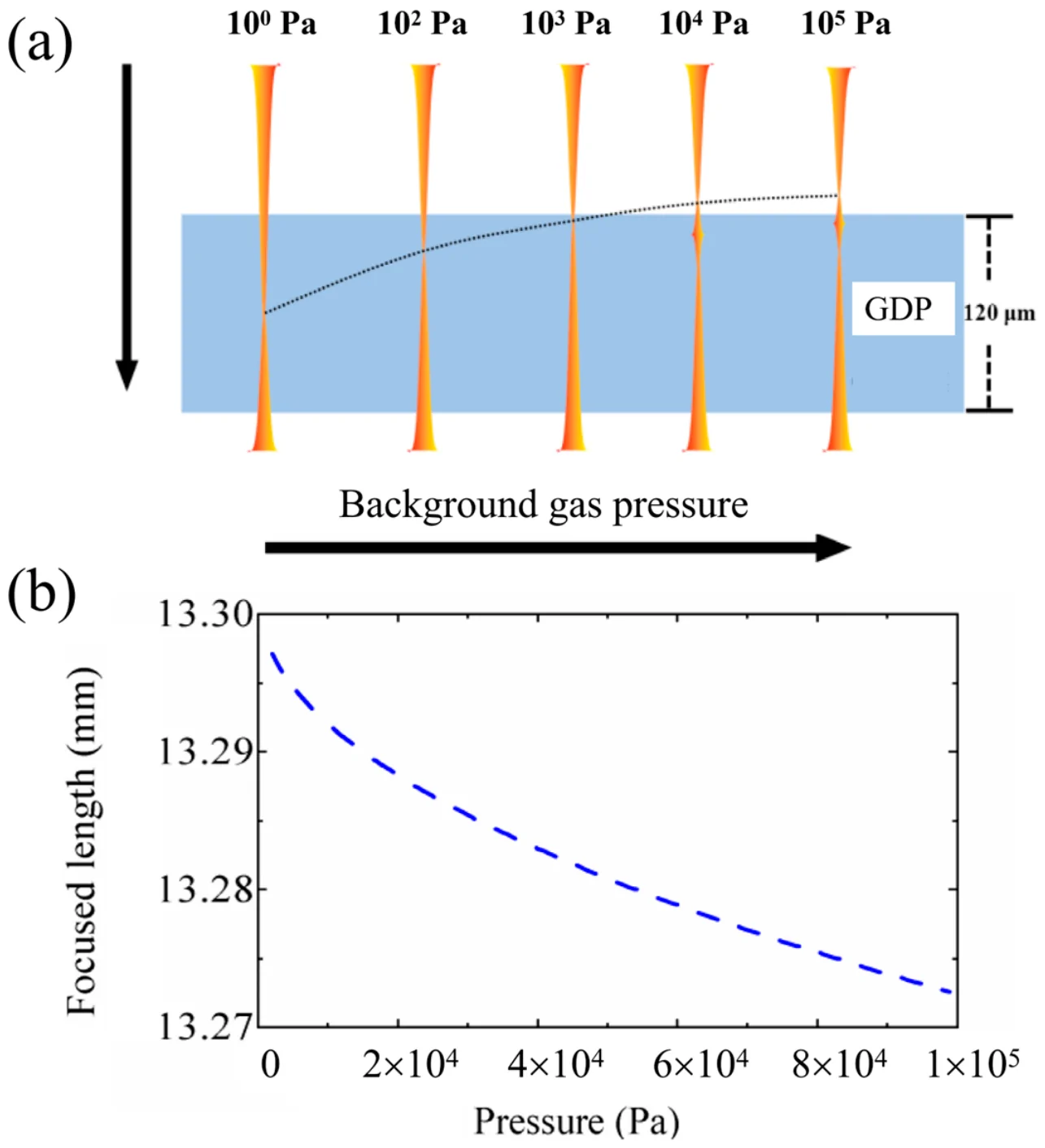
(**a**) Schematic illustration of pressure-dependent shifts in the effective femtosecond laser focal position relative to the GDP film. (**b**) The calculated modified laser collapse focusing distance as a function of ambient gas pressure.

The non-monotonic trend in entrance diameter suggests that pressure-dependent nonlinear laser propagation governs the surface spot size. A schematic diagram of focal positions at different background pressures is shown in Figure 6a. The laser self-focusing collapse distance is calculated using the following equation [21]:

(2) $$L_{c}=\frac{0.367k_{0}r_{0}^{2}}{\sqrt{{\left(\sqrt{P_{in}/P_{cr}}-0.852\right)}^{2}-0.0219}}$$

in which $P_{cr}=3.77\lambda_{0}^{2}/8\pi n_{0}n_{2}$ is the power of the pulsed laser, $P_{in}$ is the input power, $k_{0}$ is the wave number, r_0_ is the initial beam radius, $\lambda_{0}$ is the laser wavelength, $n_{0}$ is the linear index of refraction, and $n_{2}$ is the nonlinear index of refraction. The relation between $n_{2}$ and pressure is $n_{2}\left(p\right)=n_{2}\left(p_{0}\right)\times \tilde{p}$, $\tilde{p}=p/p_{0}$, where $p_{0}$ is the standard atmosphere and p is the actual pressure value. For a focusing laser beam, the modified collapse distance is calculated using the following equation [21]:

(3) $$\frac{1}{L_{cf}}=\frac{1}{L_{c}}+\frac{1}{f},$$

where f is the focal length of the objective lens. As shown in Figure 6b, higher pressure enhances gas-phase nonlinearity and shortens the effective collapse distance. With a fixed lens-to-sample geometry, while it is set to the middle of the GDP film thickness at low pressure, the effective focus is therefore moving upwards (Figure 6a) with increasing pressure. At moderate pressures, enhanced Kerr self-focusing can reduce the surface spot size and consequently the entrance diameter [40,41,42]. At higher pressures, stronger ionization and plasma-induced defocusing or refocusing can redistribute the beam energy and reverse this trend. The monotonic decrease in exit diameter with increasing pressure is consistent with the combined effects of focal-position shifts and plasma shielding: as the focal position moves closer to the entrance surface, the plasma/plume is generated at the solid–gas interface, where it experiences weaker confinement than within the bulk material. Nevertheless, its expansion into the ambient gas produces pronounced shielding, which reduces the effective laser energy delivered to the bottom of the hole. Furthermore, the gas composition in the vacuum chamber under our pressure is dominated by N_2_ and O_2_, which the nonlinear propagation effects of the laser beam in these two neutral gases should show no appreciable difference. These observations indicate that pressure-dependent nonlinear propagation and plasma dynamics jointly determine the final hole geometry.

### 3.3. Effect of Ambient Pressure on Drilling Efficiency

**Figure 7:**
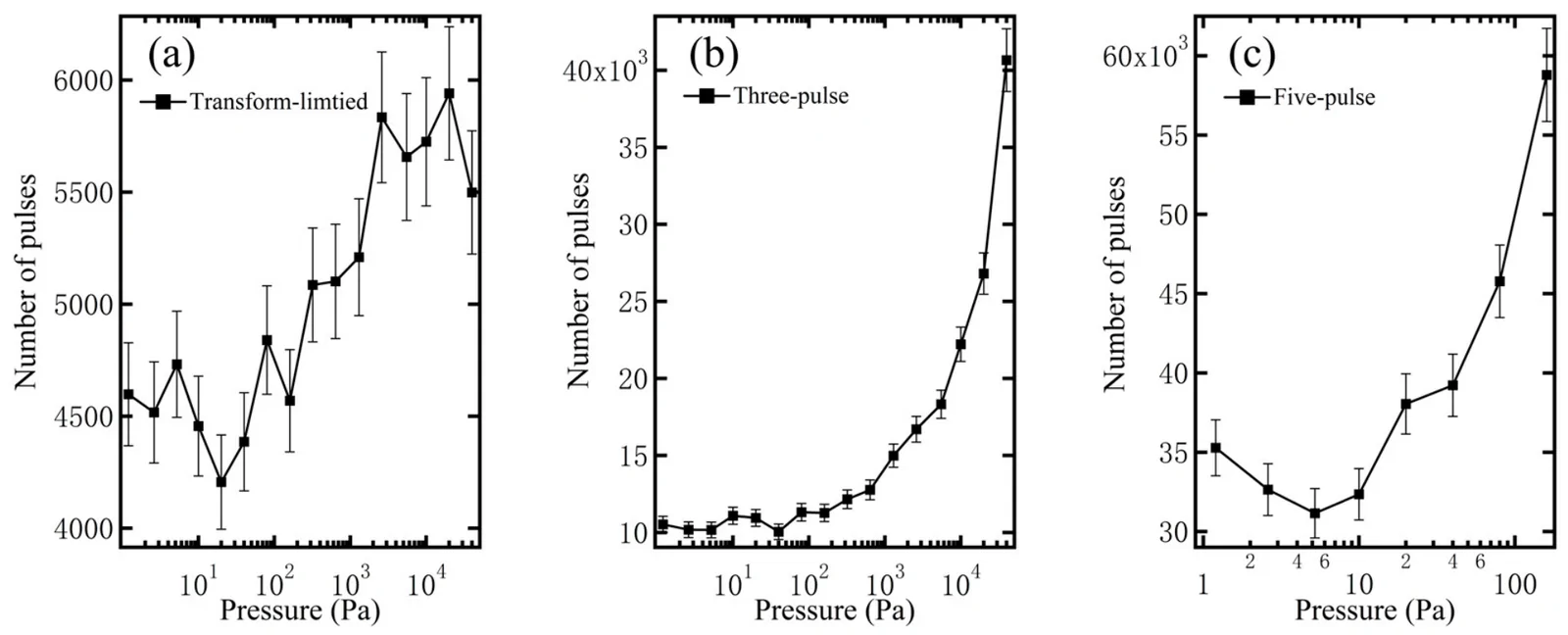
Number of laser pulses required to achieve breakthrough as a function of ambient gas pressure for (**a**) transform-limited pulses, (**b**) three-pulse sequences, and (**c**) five-pulse sequences. All measurements were performed six times under each condition.

Figure 5 presents the number of laser pulses required to achieve breakthrough as a function of ambient pressure by using three different pulse shapes. The results reveal a pronounced overall decrease in drilling efficiency with increasing pressure. For transform-limited pulses, approximately 4600 pulses are required at 1.4 Pa, whereas about 5900 pulses are required at 4 × 10^4^ Pa as can be seen in Figure 7a. For the three-pulse sequence shown in Figure 7b, the breakthrough pulse count increases more rapidly with pressure and reaches approximately 4 × 10^4^ pulses in the high-pressure regime. The five-pulse sequence does not produce through-holes when the pressure exceeds ~4 × 10^2^ Pa, so there is no data after this pressure in Figure 7c. These trends are consistent with pressure-dependent plume and plasma confinement. The ablation plasma is not cooled rapidly compared with the atmospheric pressure condition and does not expand too much compared with the vacuum condition [27]. At low pressure, rapid expansion of the ablation products reduces the residence time of dense ionized and particulate material near the surface, thereby limiting plasma shielding and allowing subsequent pulses to couple more directly to the sample.

At higher pressures, the surrounding gas increasingly confines the ablation plume and prolongs the presence of dense excited and ionized material near the interaction region [23,24,25,26,27]. Under atmospheric pressure conditions, expansion of ablation plasma is strongly limited and the cooling speed of plasma is very high, and a more pronounced confinement effect of the plasma takes place. This near-surface layer can attenuate the incident laser through absorption and scattering, reducing the effective energy delivered to the material and increasing the number of pulses required for breakthrough. Pulse shaping further modifies this behavior. With a subpulse delay of 0.3 ps, successive subpulses arrive while the laser-excited electron population and the incipient plasma are still evolving. Such temporal overlap can enhance energy deposition under favorable conditions, but it can also strengthen plasma shielding when the plume is strongly confined. Under the conditions in the present study, the latter effect becomes increasingly significant as both ambient pressure and the number of subpulses increase, accounting for the pronounced efficiency loss observed for the multi-pulse sequences at high pressure.

Overall, the ambient pressure affects GDP drilling through several coupled mechanisms. First, it controls the transport and redeposition of ablation products by modifying the gas-phase collision rate and plume confinement, thereby determining surface cleanliness. Second, pressure-dependent nonlinear propagation changes the effective focal position and surface spot size, which strongly influences the entrance diameter. Third, plume and plasma confinement alter plasma shielding and the fraction of laser energy that reaches the deeper regions of the evolving hole. The balance among these mechanisms determines the final morphology and breakthrough pulse count. The investigated parameter space, a pressure range around 10^2^~10^3^ Pa, combined with a three-pulse sequence offers the best compromise between higher surface quality, small hole diameter, low taper, and high drilling efficiency. The studied influence of focusing and plasma on laser ablation can be extended to future development by using shaped laser beams with shorter wavelengths [43,44]. Unlike prior femtosecond laser ablation studies on polymers that typically optimize single variables under fixed ambient conditions, the present work introduces a dual-parameter strategy that systematically couples backing-pressure regulation with tailored temporal pulse trains specifically for glow discharge polymer films. Moreover, we reveal a non-monotonic pressure dependence of the entrance diameter—contrary to the monotonic trends generally assumed in the literature—and provide a mechanistic interpretation based on pressure-induced nonlinear propagation and plasma shielding, thereby establishing a new operational framework for precision micromachining of polymer films.

## 4. Conclusions

We have systematically investigated the effects of ambient pressure and pulse shaping on femtosecond laser drilling of GDP films. Lowering the ambient pressure markedly suppresses debris redeposition and improves surface cleanliness. The entrance diameter varies non-monotonically with pressure, whereas the exit diameter decreases continuously as pressure increases, leading to larger entrance–exit diameter differences at high pressure. Among the tested pulse configurations, the three-pulse sequence provides the most favorable dimensional uniformity over a broad pressure range; at 2.8 Pa, the entrance–exit diameter difference is approximately 0.3 μm. A low-pressure environment also reduces the breakthrough pulse count by mitigating plume confinement and plasma shielding. These results demonstrate that ambient pressure and temporal pulse shape must be optimized jointly: low pressure enhances debris removal and energy delivery, while an appropriate pulse sequence reduces taper without the severe efficiency loss observed for the five-pulse sequence at elevated pressure. These findings establish a practical basis for high-quality and high-efficiency microhole fabrication in GDP films and related transparent polymer materials.

## Data Availability

The original contributions presented in this study are included in the article. Further inquiries can be directed to the corresponding authors.
